# Supplementary material for: Production risk and technical efficiency of dry-season vegetable farmers in the Upper East Region of Ghana
Source: PLoS One. 2025 Feb 13;20(2):e0309375. doi: 10.1371/journal.pone.0309375 (PMC11824998; doi:10.1371/journal.pone.0309375)
Supplement: S1 File — (DOCX) [file pone.0309375.s002.docx]

# Regression Results

1. **RESULTS OF THE TRAN-LOG FRONTIER REGRESSION**

|  | (1) | (2) | (3) | (4) |
| --- | --- | --- | --- | --- |
| VARIABLES | Frontier | Mu | Usigma | Vsigma |
|  |  |  |  |  |
| lSNLabourC | 0.434*** |  |  |  |
|  | (0.0416) |  |  |  |
| lSNvegseedc | 0.0988*** |  |  |  |
|  | (0.0383) |  |  |  |
| lSNFertcost | 0.384*** |  |  |  |
|  | (0.0482) |  |  |  |
| lSNAgrochemC | 0.321*** |  |  |  |
|  | (0.0309) |  |  |  |
| lSNTirrigatnC | 0.105*** |  |  |  |
|  | (0.0322) |  |  |  |
| lSNLabourC2 | 0.195 |  |  |  |
|  | (0.120) |  |  |  |
| lSNvegseedc2 | -0.156 |  |  |  |
|  | (0.102) |  |  |  |
| lSNFertcost2 | -0.0855 |  |  |  |
|  | (0.0947) |  |  |  |
| lSNAgrochemC2 | 0.0546 |  |  |  |
|  | (0.0352) |  |  |  |
| lSNTirrigatnC2 | 0.383*** |  |  |  |
|  | (0.0761) |  |  |  |
| lSNLabourClSNvegseedc | -0.114 |  |  |  |
|  | (0.0736) |  |  |  |
| lSNLabourClSNFertcost | 0.00880 |  |  |  |
|  | (0.0941) |  |  |  |
| lSNLabourClSNAgrochemC | -0.0400 |  |  |  |
|  | (0.0523) |  |  |  |
| lSNLabourClSNTirrigatnC | -0.238*** |  |  |  |
|  | (0.0680) |  |  |  |
| lSNvegseedclSNFertcost | 0.275*** |  |  |  |
|  | (0.0680) |  |  |  |
| lSNvegseedclSNAgrochemC | 0.0455 |  |  |  |
|  | (0.0533) |  |  |  |
| lSNvegseedclSNTirrigatnC | -0.140** |  |  |  |
|  | (0.0633) |  |  |  |
| lSNFertcostlSNAgrochemC | 0.0517 |  |  |  |
|  | (0.0563) |  |  |  |
| lSNFertcostlSNTirrigatnC | -0.0712 |  |  |  |
|  | (0.0606) |  |  |  |
| lSNAgrochemClSNTirrigatnC | -0.0351 |  |  |  |
|  | (0.0405) |  |  |  |
| vegirrno |  |  | 0.00141 |  |
|  |  |  | (0.00355) |  |
| gravityIrrigtn |  |  | -1.415*** |  |
|  |  |  | (0.510) |  |
| pumpIrrigtn |  |  | -0.872** |  |
|  |  |  | (0.369) |  |
| o.handIrrigtn |  |  | 5.29e-11 |  |
|  |  |  | (0) |  |
| Gender |  |  | 0.623 |  |
|  |  |  | (0.428) |  |
| Age |  |  | 0.0183 |  |
|  |  |  | (0.0604) |  |
| Age1 |  |  | 0.000197 |  |
|  |  |  | (0.000661) |  |
| HhSize |  |  | 0.0945*** |  |
|  |  |  | (0.0325) |  |
| FExp |  |  | -0.0762*** |  |
|  |  |  | (0.0190) |  |
| Obtcredit |  |  | -0.180 |  |
|  |  |  | (0.319) |  |
| Extvisit |  |  | -0.117* |  |
|  |  |  | (0.0683) |  |
| pepper |  |  | -0.398 |  |
|  |  |  | (0.285) |  |
| onion |  |  | -0.179 |  |
|  |  |  | (0.328) |  |
| tomato |  |  | 0.450 |  |
|  |  |  | (0.328) |  |
| GE |  |  | -0.178 |  |
|  |  |  | (0.483) |  |
| 1.educ |  |  | 0.118 |  |
|  |  |  | (0.280) |  |
| 2.educ |  |  | 0.182 |  |
|  |  |  | (0.413) |  |
| 3.educ |  |  | -0.815 |  |
|  |  |  | (0.496) |  |
| LabourC |  |  |  | -0.000643*** |
|  |  |  |  | (0.000186) |
| vegseedc |  |  |  | -0.000135 |
|  |  |  |  | (0.00138) |
| Fertcost |  |  |  | 0.00227** |
|  |  |  |  | (0.00104) |
| AgrochemC |  |  |  | -0.00281** |
|  |  |  |  | (0.00125) |
| TirrigatnC |  |  |  | 0.000177 |
|  |  |  |  | (0.000365) |
| Constant | 0.184*** | -0.0740 | -2.212 | -1.828*** |
|  | (0.0420) | (0.166) | (1.503) | (0.285) |
|  |  |  |  |  |
| Observations | 322 | 322 | 322 | 322 |
