# Supplementary material for: Production risk and technical efficiency of dry-season vegetable farmers in the Upper East Region of Ghana
Source: PLoS One. 2025 Feb 13;20(2):e0309375. doi: 10.1371/journal.pone.0309375 (PMC11824998; doi:10.1371/journal.pone.0309375)
Supplement: Attachment — Submitted filename: RESPONSE TO REVIEWERS.docx [file pone.0309375.s003.docx]

**Dear academic editor and reviewers,**

**RESPONSE TO REVIEWERS**

After reading through your comments and suggestions, we write to respond to your concerns on the paper as follows:

**A. ACADEMIC EDITOR**

1. The manuscript has been revised to meet PLOS ONE’s style requirements

<https://journals.plos.org/plosone/s/file?id=wjVg/PLOSOne_formatting_sample_main_body.pdf> and

<https://journals.plos.org/plosone/s/file?id=ba62/PLOSOne_formatting_sample_title_authors_affiliations.pdf>

2. The entire manuscript has been thoroughly copyedited for language usage, spelling, and grammar.

3. There is a statement provided in the methodology section that briefly explains why there was no need for a formal field site access permit for the study.

4. A statement regarding the ethical approval for the study has also been provided right after the methods section.

5. These two references have been deleted and the most current and relevant references replaced accordingly.

6. Figure 1, the map of the study area has been deleted

**B. REVIEWER 1**

**ABSTRACT**

1. The abstract has been revised to conform to the suggestions.

**INTRODUCTION**

The entire introduction has also been revised to:

1. Explain the triggers for food insecurity in the study area which include climate change

2. Make the motive of the study clearer,

3. Improve the justification of the study.

**METHODOLOGY**

1. Although the reviewer's concern about the methodology is unclear, we can deduce that it is about the clarity of the model estimations. The conventional technical efficiency model is given as follows:

$Y_{i}=f\left( X_{i};\beta\right)+\varepsilon_{i}$	eqn (4)

However, the conventional model's shortcoming in capturing farmers' perceived risk behaviour led to the incorporation of the risk component into the stochastic technical efficiency model. So, one model contains both the technical efficiency (the mean output function and the technical inefficiency function) and the production risk models.

$Y_{i}=f\left( x;\beta\right)+g\left( z;\theta\right)v-q\left( w;\delta\right)u$. Equ (6)

2. Elasticity is used because, in sub-section 2.4, an explanation was given that the various inputs were normalized with land (ha) and scaled by the respective sample means. In such a situation, the first-order coefficients can be expressed as the elasticities of the technical efficiency with respect to the corresponding input cost specification.

3. The explanation of the sources of technical inefficiency is stated in the equation (6) above.

**RESULTS**

1. Frontier model is a stand-alone model that is employed to estimate technical efficiency in a production process. It does not require an average model to compare to it. In any case, the focus of the study was not to determine which model is superior in the study.

2. The input variables determine the elasticities of the production process while the socio-economic variables explain the inefficiencies of the production. The direction of the signs (either + or -) indicates whether it increases inefficiency or otherwise.

3. Also, the signs of the production risk variables tell whether the variable is risk increasing or decreasing. So, we use the sign direction of the variable to explain and not the absolute value of the variable.

**C. REVIEWER 2**

Thank you very much for your understanding of our paper. We appreciate that. However, your concern about the study's abstract has been revised, stating the inefficiency variables as you requested.

The monetary value in USD has also been stated in the table of monetary figures.

All significant independent variables have been explained, stating reasons for their significance in the results.
