# Supplementary material for: Production risk and technical efficiency of dry-season vegetable farmers in the Upper East Region of Ghana
Source: PLoS One. 2025 Feb 13;20(2):e0309375. doi: 10.1371/journal.pone.0309375 (PMC11824998; doi:10.1371/journal.pone.0309375)
Supplement: Attachment — Submitted filename: Plos one 2nd review.docx [file pone.0309375.s004.docx]

The author didn’t address all the point of revision. It was recommended that the line number need to add. But author didn’t add any number line which mislead me in revision. The authors tried to improve the grammatical error. But still, they should check it again. There is major grammatical error in the whole writing.

Abstract: 1. “Farmers in the Upper East Region …….. abysmally low.” Please remove intensified.

2. “With the present state of technology and resource constraints of farmers……” What does it mean by options? Please clarify.

Introduction: 1. Still justification and motive of the study is still unclear.

Literature: A constructive literature was recommended. Author didn’t address any literature review.

The author would benefit from the following papers:

<https://www.researchgate.net/publication/372685863_Climate_change_and_agriculture_nexus_in_Bangladesh_Evidence_from_ARDL_and_ECM_techniques>

<https://www.researchgate.net/publication/369013150_Economic_assessment_of_rice_farmers'_climate_change_adaptation_options_and_their_sustainability_a_case_of_Pabna_district_Bangladesh>

<https://www.researchgate.net/publication/356503914_Assessing_Livelihood_Adaptation_Indices_and_the_Sustainability_of_Rice_Farmers_in_Bangladesh's_Northwestern_Region>

<https://www.researchgate.net/publication/315368214_Perception_of_and_Adaptive_Capacities_to_Climate_Change_Adaptation_Strategies_and_Their_Effects_on_Rice_Production_A_Case_of_Pabna_District_Bangladesh>

Methodology:

1. The authors use production risk function to capture farm’s risk behaviour. But why did you use only technical efficiency model? Isn’t both technical and profit efficiency would calculate here to understand farmer’s behaviour in risky situation?

Result:

1. In explanation, how much the variables increase or decrease the efficiency, didn’t mention yet.

2. Which variables increase or decrease production risk mention here but still didn’t mention how much increase or decrease production risk.

3. the authors didn’t link their result to previous study.

Limitation: What are the limitations of the study? Mention the limitations.
