# Supplementary material for: Production risk and technical efficiency of dry-season vegetable farmers in the Upper East Region of Ghana
Source: PLoS One. 2025 Feb 13;20(2):e0309375. doi: 10.1371/journal.pone.0309375 (PMC11824998; doi:10.1371/journal.pone.0309375)
Supplement: Attachment — Submitted filename: RESPONSE TO REVIEWER 2.docx [file pone.0309375.s005.docx]

**Dear academic editor and reviewer,**

**RESPONSE TO REVIEWERS**

After reading through your comments and suggestions, we write to respond to your concerns on the paper as follows:

**A. ACADEMIC EDITOR**

1. The manuscript has been revised to meet PLOS ONE’s style requirements

<https://journals.plos.org/plosone/s/file?id=wjVg/PLOSOne_formatting_sample_main_body.pdf> and

<https://journals.plos.org/plosone/s/file?id=ba62/PLOSOne_formatting_sample_title_authors_affiliations.pdf>

**B. REVIEWER 2**

**ABSTRACT**

1. The abstract has been revised to conform to the suggestions. Farmers in the Upper East Region …….. abysmally low.” Intensified has been deleted.
2. Clarification on the statement “With the present state of technology and resource constraints of farmers……” has been clarified.

**INTRODUCTION**

The entire introduction has also been revised to:

1. Explain the move for the study in the last paragraph.
2. Provide a constructive literature review

**METHODOLOGY**

1. Yes, the study indeed employed the production risks model incorporated into the technical efficiency model to capture the farm’s risk behavior. The profit efficiency model was not included in the calculation because farm-level profits were not calculated in the analysis. The value of output in monetary terms was an aggregation for the dependent variable since each farmer produces more than one vegetable in a season. This is consistent with the literature that states that “technical efficiency studies on the products of the whole farm, rather than specific products usually apply monetary value instead of physical quantities” [56, 95-99].

**RESULTS**

1. The input variables determine the elasticities of the production process.

2. The socio-economic variables explain the inefficiencies of the production process where the direction of the signs (either + or -) indicates whether it increases or decreases the inefficiency.

3. Also, the positive (+) signs of the production risk variables tell whether the variable is risk increasing or negative (-) signs show risk decreasing. So, we use the sign direction of the variable to explain and not the absolute value of the variable.

4. The results section has been revised to link the explanation to the literature.

The limitations of the study

The data on the irrigation of the vegetables should have been in terms of the volume of water applied per day instead of the frequency of applications per season. This does not tell the exact quantity of water requirement per farm size (ha).
